# Phase 1A/1B dose-escalation and -expansion study to evaluate the safety, pharmacokinetics, food effects and antitumor activity of pamiparib in advanced solid tumours

**DOI:** 10.1038/s41416-021-01632-2

**Published:** 2021-11-18

**Authors:** Jason D. Lickliter, Mark Voskoboynik, Linda Mileshkin, Hui K. Gan, Ganessan Kichenadasse, Kathy Zhang, Maggie Zhang, Zhiyu Tang, Michael Millward

**Affiliations:** 1 Nucleus Network, Melbourne, VIC Australia; 2 Central Clinical School, Monash University, Melbourne, VIC Australia; 3 Peter MacCallum Cancer Centre-East Melbourne, East Melbourne, VIC Australia; 4 Olivia Newton-John Cancer Wellness and Research Centre, Austin Hospital, Heidelberg, Melbourne, VIC Australia; 5 La Trobe University School of Cancer Medicine, Heidelberg, VIC Australia; 6 Department of Medicine, University of Melbourne, Heidelberg, VIC Australia; 7 Flinders Centre for Innovation in Cancer, Flinders Medical Centre, Bedford Park, SA Australia; 8 BeiGene USA, Inc., San Mateo, CA USA; 9 Linear Clinical Research & University of Western Australia, Nedlands, WA Australia

**Keywords:** Ovarian cancer, Drug development

## Abstract

**Background:**

Pamiparib, a PARP1/2 inhibitor, demonstrated antitumor activity in preclinical models.

**Methods:**

This Phase 1A/1B dose-escalation/dose-expansion study enrolled adults (≥18 years) with advanced/metastatic cancer. The dose-escalation phase evaluated the recommended Phase 2 dose (RP2D), maximum tolerated dose (MTD), and pharmacokinetics; the dose-expansion phase evaluated the antitumor activity and food effects.

**Results:**

Patients (*N* = 101) were enrolled in dose-escalation (*n* = 64) and dose-expansion (*n* = 37). During BID dose-escalation, dose-limiting toxicities were Grade 2 nausea (*n* = 1, 40 mg; *n* = 1, 80 mg); Grade 2 nausea and Grade 2 anorexia (*n* = 1, 120 mg), Grade 2 nausea, Grade 3 fatigue and Grade 3 paraesthesia (*n* = 1, 120 mg); MTD was 80 mg BID and RP2D was 60 mg BID. Common adverse events (AEs) were nausea (69.3%), fatigue (48.5%) and anaemia (35.6%); the most common Grade ≥3 AE was anaemia (24.8%). There was a dose-proportional increase in pamiparib exposure; no food effects on pharmacokinetics were observed. In the efficacy-evaluable population (*n* = 77), objective response rate (ORR) was 27.3% (95% CI, 17.7–38.6%). Median duration of response was 14.9 months (95% CI, 8.7–26.3). In the epithelial ovarian cancer (EOC)-evaluable population (*n* = 51), ORR was 41.2% (95% CI, 27.6–55.8%).

**Conclusions:**

Pamiparib was tolerated with manageable AEs, and antitumor activity was observed in patients with EOC.

**ClinicalTrials.gov Identifier:**

NCT02361723.

## Background

Poly(ADP-ribose) polymerase 1 and 2 (PARP1/2) proteins play a central role in the regulation of the nuclear processes of DNA repair, genome stability, and programmed cell death [1, 2]. The main function of PARP proteins is to detect single-strand breaks in DNA and target them for repair [1]. In normal cells, double-strand DNA breaks are repaired by homologous recombination; however, this repair mechanism is compromised in the presence of loss-of-function mutations in the tumour suppressor genes, *BRCA1* and *BRCA2* [1]. Inhibition of PARP proteins allows for the accumulation of unrepaired single-strand breaks, which are converted to double-strand breaks during cell division and can lead to apoptosis/cell death [1]. Loss of *BRCA1/2* function leads to inhibition of homologous recombination-mediated repair of double-strand DNA breaks, which renders cells highly susceptible to DNA lesions caused by PARP inhibition. Currently, several mechanisms have been proposed to explain how PARP inhibition leads to cell death, including modulation of the PARylation activity of PARP and PARP–DNA trapping [3].

PARP inhibitors are a class of therapeutic agents that have been shown to be effective for the treatment of malignancies, including tumours associated with *BRCA1/2* mutations or without *BRCA* mutations but with homologous recombination deficiencies [3–5]. Pamiparib is an oral, potent, and selective PARP1/2 inhibitor that has shown PARP–DNA complex trapping and inhibition of PARylation, antitumor activity, and brain penetration in preclinical models [6, 7]. Specifically, pamiparib showed potent PARP–DNA complex trapping and antiproliferative activities against cell lines harbouring *BRCA* gene mutations or homologous recombination deficiencies (HRD), as well as a time-dependent and dose-dependent inhibition of PARylation in breast cancer xenografts [6]. In addition, pamiparib induced significant tumour regression in a *BRCA1*-mutant breast cancer xenograft model with 16-fold higher efficacy compared with olaparib [6]. Taken together, these nonclinical results suggest that pamiparib could offer clinical benefits to patients with tumours harbouring *BRCA* mutations or HRD deficiencies. In addition, acquired resistance to PARP inhibitors, which may result from a PARP inhibitor being a substrate of P-gp (P-glycoprotein) and BCRP (breast cancer resistance protein) [8–10], has been reported to occur in most patients with advanced cancer who have received this class of agents [11]. Pamiparib is not a substrate of P-glycoprotein or of BCRP [6], and these characteristics may prevent the acquired resistance that has been reported to occur with other PARP inhibitors [12]. Results of the current study and of a Phase 1 study in patients with ovarian cancer have shown that the bioavailability of pamiparib is high, with near-complete absorption in humans [13].

Here, we present results of a first-in-human (FIH) dose-escalation/dose-expansion study (NCT02361723), which assessed outcomes of pamiparib in patients with advanced solid tumours. The primary objectives of this study were to evaluate the safety and tolerability of pamiparib, including determining the maximum tolerated dose (MTD) and the recommended Phase 2 dose (RP2D). Secondary objectives were to characterise the pharmacokinetics (PK) and food effects of pamiparib and to evaluate antitumor response.

## Methods

### Study design

**Fig. 1:**
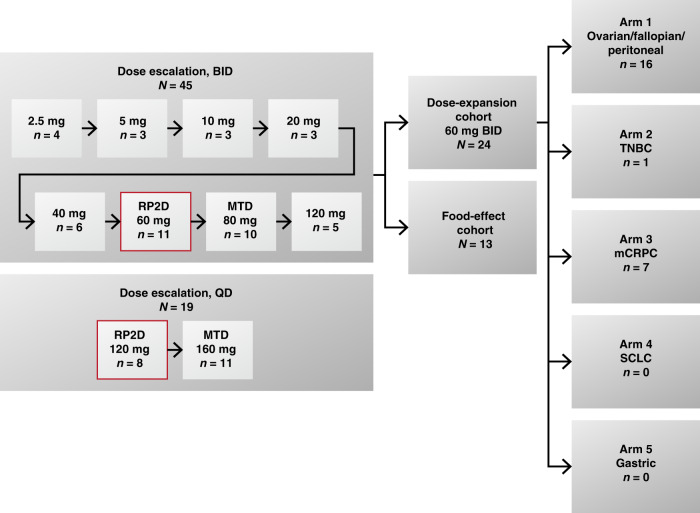
Study design. BID twice daily, mCRPC metastatic castration-resistant prostate cancer, MTD maximum tolerated dose, QD once daily, RP2D recommended Phase 2 dose, SCLC small-cell lung cancer, TNBC triple-negative breast cancer.

This was an open-label, Phase 1, dose-escalation/dose-expansion study, conducted across six study centres in Australia. The dose-escalation phase evaluated twice-daily (BID) and once-daily (QD) dosing, and the dose-expansion phase evaluated the BID RP2D from the dose-escalation phase and food effects (Fig. 1). The food-effects cohort comprised two sequences: fast followed by fed (sequence 1) and fed followed by fast (sequence 2). There were eight BID dose-escalation cohorts (2.5–120 mg) and two QD dose-escalation cohorts (120 mg and 160 mg), with ≥3 patients enrolled at each dose level. The decision to proceed to the next dose cohort was determined by the Safety Monitoring Committee and followed a modified 3 + 3 escalation scheme. Dose escalation continued until ≥2 dose-limiting toxicities (DLTs, criteria listed in Supplementary Table S1) were observed in a cohort of three to six patients. When ≥2 DLTs occurred in the first 23 days of a dose level, the next lower dose level was declared the MTD. Safety was monitored and DLTs were assessed in all dose cohorts. During BID dose expansion, patients with selected tumour types were enrolled into one of five arms to further evaluate pamiparib 60 mg BID (RP2D determined from BID dose escalation). The food-effects cohort investigated the effects of food on the PK of pamiparib in patients with advanced solid tumours. The first patient commenced treatment on July 3, 2014 and the study was completed on September 3, 2019.

### Patient population

Patients enrolled in the dose-escalation phase and in the dose-expansion phase (including the food-effects cohort) were adults, aged ≥18 years, with histologically or cytologically confirmed advanced or metastatic cancer for which no effective standard therapy was available; neither germline nor somatic *BRCA1/2* mutations (*gBRCA*^mut^ or *sBRCA*^mut^) were required for enrolment (except in Arm 1, Arm 2 and Arm 3 of the dose-expansion phase). Although *BRCA* mutation status was not required for enrolment into the dose-escalation cohorts, it was recommended that blood samples be collected at the screening to assess germline *BRCA* mutation status. Patients in Arm 1, Arm 2 and Arm 3 were required to have either HRD or *BRCA1/2* mutation status for enrolment into the dose-expansion phase.

Patients enrolled in the dose-expansion phase had histologic or cytologic confirmation of malignancy that had progressed to the advanced or metastatic state or was stage IV at diagnosis. Eligible patients had measurable disease per Response Evaluation Criteria in Solid Tumours (RECIST) v1.1 and had received one or more prior chemotherapy regimens in the advanced or metastatic setting. Patients were recruited to one of five expansion arms. Patients enrolled in Arm 1 were required to have platinum-sensitive, high-grade epithelial ovarian cancer (EOC; included ovarian, fallopian or primary peritoneal cancer) with either known deleterious or suspected deleterious *gBRCA*^mut^ or *sBRCA*^mut^, or HRD-positive status as assessed using the Myriad myChoice® diagnostic test. If *BRCA* mutation or HRD status was unknown at the time of enrolment, then archival tissue was required for analysis; relevant blood and/or tumour samples were collected for assessment of *BRCA* mutation and HRD status. Patients with EOC who did not have measurable disease based on RECIST v1.1 were considered eligible if their disease was evaluable based on Gynecologic Cancer Intergroup CA-125 response criteria. Patients with EOC were required to have received ≥1 line of platinum-containing therapy and must not have progressed or have had recurrent disease within 6 months of completing the last platinum-containing regimen. Arm 2 included patients with triple-negative breast cancer (TNBC), Arm 3 included patients with metastatic castration-resistant prostate cancer (mCRPC), Arm 4 was a cohort to enrol patients with extensive-stage small-cell lung cancer (SCLC) and Arm 5 was a designated cohort to include patients with gastric cancer (GC). Additional inclusion and exclusion criteria are presented in the Supplemental Appendix.

### Treatment administration

Patients in the dose-escalation phase received a single oral dose of pamiparib on day 1 of cycle 1 to collect samples for single-dose PK assessment over 48 h, followed by continuous daily doses (BID and QD) starting on day 3 of cycle 1 as a 21-day period of repeated drug administration (days 3–23), and then continued every 21-day cycle thereafter until disease progression, toxicity, or patient withdrawal. Eight BID dose levels (2.5, 5, 10, 20, 40, 60, 80, 120 mg) were administered (Fig. 1). After completion of BID dose-escalation, alternative dosing regimens of 120 and 160 mg QD were selected for exploration based on the total daily dose equivalent to the RP2D (60 mg BID) and MTD (80 mg BID), respectively. The QD dosing regimen was added as an amendment to the protocol with the purpose of exploring the feasibility of a more convenient regimen. During the dose-expansion phase, patients received pamiparib 60 mg BID (RP2D from BID dose-escalation phase) continuously from day 1 of cycle 1.

Patients in the food-effects cohort received a single dose of pamiparib 60 mg (based on the RP2D) on day 1 of cycle 1, followed by a 5-day washout; patients then received another single dose of 60 mg on day 6. Seven patients in sequence 1 (fast followed by fed) received pamiparib after a ≥ 10-h fast on day 1 and after consuming a standard high-fat meal (described in the Supplemental Appendix) 30 min prior to drug administration on day 6. Six patients in sequence 2 (fed followed by fast) received pamiparib after a high-fat meal on day 1 and after a ≥ 10-h fast on Day 6. From day 8 of cycle 1 onward, patients received 60-mg BID treatment.

### Assessments

Safety and tolerability were assessed throughout the study by monitoring adverse events (AEs), serious AEs, clinical laboratory measurements and physical examinations. Adverse events were categorised according to their severity (National Cancer Institute Common Terminology Criteria for Adverse Events v4.03) and relationship to the study treatment.

Pamiparib in plasma was measured using a validated liquid chromatography-tandem–mass spectrometry assay with a lower limit of quantification of 1.0 ng/mL. The collection schedule for blood samples used to assess the PK profile is presented in the Supplemental Appendix. Summary PK parameters, including area under the concentration-time curve from time 0 to 9 h (AUC_0-9_) or infinity (AUC_0-inf_), maximal plasma concentration (*C*_max_) and time to maximal plasma concentration (*T*_max_), were estimated. The pharmacodynamic (PD) activity of pamiparib was explored through the evaluation of poly(ADP-ribose [PAR]) formation in peripheral blood mononuclear cells (PBMCs) after pamiparib administration. The schedule for blood sample collection (PK and PBMCs) and additional assessment information for PAR levels in PBMCs and pharmacodynamic activity is presented in the Supplemental Appendix. Antitumor activity was assessed by radiographic imaging (computed tomography or magnetic resonance imaging) during screening, within 28 days of first pamiparib dose, every 6 weeks in the first 12 months and every 9 weeks thereafter.

Tumour response was evaluated by RECIST v1.1. For patients with ovarian cancer, tumour response was assessed per RECIST v1.1 and by Gynecologic Cancer Intergroup (GCIG) CA-125 criteria. Tumour response for patients with prostate cancer was assessed by Prostate Cancer Clinical Trials Working Group 2 (PCWG2) criteria. Objective response rate (ORR) was defined as the proportion of patients achieving a confirmed complete response (CR) or partial response (PR) on study treatment. Blood tumour antigens (e.g., carcinoembryonic antigen for colorectal cancer, CA-125 for ovarian cancer and prostate-specific antigen [PSA] for mCRPC) were assessed during screening, every 6 weeks after the first pamiparib dose in the first 12 months, and every 9 weeks thereafter.

Analyses of ORR (95% confidence interval [CI]) by investigator assessment were performed using predefined subgroups of the EOC population, including age group (<65 years versus ≥65 years), baseline ECOG performance status (0 versus 1), solid tumour stage (Stage III versus IV), *BRCA*/HRD status (germline *BRCA* mutation versus wild-type or unknown; germline or somatic *BRCA* mutation versus wild-type or unknown; HRD-positive versus negative or unknown), and platinum response status (platinum-sensitive versus platinum-resistant versus platinum-refractory).

### Statistical methods

The safety population comprised all patients in the dose-escalation and dose-expansion phases who received at least one dose of pamiparib (Supplementary Table S2). Patients in the safety analysis set for whom valid pamiparib PK parameters were estimated and for whom evaluable PD results were available represented the PK population and the PD population, respectively. The efficacy-evaluable population included patients in the safety analysis set who had at least one evaluable postbaseline tumour assessment or those who discontinued due to clinical disease progression or early death before tumour assessment could be performed. The EOC efficacy-evaluable population was a subset of the overall efficacy-evaluable population that included patients with EOC from both the dose-escalation and dose-expansion phases. Patients in the dose-expansion phase without measurable disease at baseline per RECIST v1.1 were excluded from the efficacy-evaluable population. Patients with EOC who had a pretreatment sample within 2 weeks prior to the first dose date that was at least twice the upper limit of the reference range were included in the CA-125–evaluable population. The PSA-evaluable population was comprised of patients with mCRPC who had a baseline PSA sample prior to the first dose date and at least one postbaseline PSA sample before the date of the new anticancer treatment.

An estimated 65 patients were planned for dose escalation (BID, *n* = 45; QD, *n* = 20). In the dose-expansion phase, it was anticipated that ~20 patients would be enrolled in each arm to explore preliminary signals of clinical efficacy and to confirm the safety and tolerability of pamiparib in patients with EOC, TNBC, mCRPC, SCLC and GC; however, the planned analysis was revised and antitumor activity was not assessed individually in Arms 2 (TNBC), 4 (SCLC) and 5 (GC) as the expansion arms were terminated due to slow enrolment. Despite this revision in the planned analysis, the primary objective was still achieved in the dose-escalation portion by identifying the RP2D.

Descriptive statistics were used to summarise all study data (see Supplemental Appendix). Progression-free survival (PFS) and event-free rates were estimated using the Kaplan–Meier method along with the corresponding 95% CI. Pharmacokinetic parameters were derived using standard non-compartmental methods with Phoenix WinNonlin Version 6.4 or higher (Pharsight Corp., Mountain View, California).

## Results

### Disposition and baseline disease characteristics

Across the total dose-escalation population (*n* = 64), the dose-expansion cohort (*n* = 24) and the food-effects cohort (*n* = 13), 101 patients were enrolled and all patients received at least one dose of pamiparib (Fig. 1). In the dose-expansion cohort (*n* = 24), Arm 1 (EOC) enrolled 16 patients, Arm 2 (TNBC) enrolled one patient, and Arm 3 (mCRPC) enrolled seven patients; Arm 4 (SCLC) and Arm 5 (GC) did not enrol patients. Arms 2, 4 and 5 were terminated due to slow enrolment. As of September 3, 2019 (data cut-off), the median study follow-up was 5.5 months (range, 0.4–57.1); all patients had discontinued study treatment due to progressive disease (54.5%), investigator’s decision (8.9%), AE (5.9%), withdrawal of consent (4.0%), or for ‘other’ reasons (26.7%). The ‘other’ category included 19 patients with clinical progression, seven patients who continued pamiparib after study closure (transferred to a compassionate use study), and one patient who chose not to continue study treatment.

In the overall dose-escalation and dose-expansion populations, most patients were female (79%) and white (90%) (Table 1). The median age was 60 years (range, 37–83) and 67% of patients were <65 years of age. The median time from the initial diagnosis to study entry was 3.22 years (range, 0.4–22.1). The most common types of solid tumour were ovarian (55.4%), prostate (11.9%) and breast (6.9%); all other solid tumour types occurred in ≤5% of the total patients. A total of 63 patients from across all dose-escalation (BID and QD) and dose-expansion arms were included in the EOC subgroup (ovarian, *n* = 56; fallopian tube, *n* = 5; peritoneum, *n* = 2) (Supplementary Table S3). Approximately half of the patients with EOC had tumours with *gBRCA*^*mut*^ or *sBRCA*^*mut*^ (49.2%), about one-quarter had wild-type *BRCA* (23.8%), and about one-quarter had tumours with unknown *BRCA* status (27.0%). A total of 54% patients with EOC had HRD-positive tumours. In the total EOC population (*N* = 63), 19.0% of patients were platinum-refractory, 41.3% platinum-resistant and 39.7% platinum-sensitive. All patients received at least one prior therapy; the median number of prior regimens was three (range, 1–15). Although baseline characteristics were generally balanced between the BID (*n* = 27) and QD (*n* = 9) cohorts of the EOC population, patients were slightly older in the latter cohort. In the BID and QD cohorts, respectively, the median age was 59 years (range, 40–71) and 64 (range, 53–72) years and 74.1% and 55.6% were <65 years of age.

**Table 1:** Patient demographics and baseline characteristics in the total dose-escalation and dose-expansion populations.

	Dose escalation (*n* = 64)	Dose expansion (*n* = 37)	Total (*N* = 101)
Sex, *n* (%)			
Female	51 (79.7)	29 (78.4)	80 (79.2)
Male	13 (20.3)	8 (21.6)	21 (20.8)
Age, years			
Median (range)	59.5 (37–83)	60.0 (37–81)	60.0 (37–83)
Group, *n* (%)			
<65	42 (65.6)	26 (70.3)	68 (67.3)
≥65	22 (34.4)	11 (29.7)	33 (32.7)
ECOG status, *n* (%)			
0	22 (34.4)	15 (40.5)	37 (36.6)
1	41 (64.1)	22 (59.5)	63 (62.4)
2^a^	1 (1.6)	0 (0)	1 (1.0)
Race, *n* (%)			
Asian	6 (9.4)	2 (5.4)	8 (7.9)
White	57 (89.1)	34 (91.9)	91 (90.1)
Other	1 (1.6)	1 (2.7)	2 (2.0)
Median time from initial diagnosis to study entry, year (range)	3.38 (0.4–22.1)	2.80 (0.6–20.4)	3.22 (0.4–22.1)
Type of solid tumour, *n* (%)			
Adenocarcinoma or cancer unknown primary^b^	2 (3.1)	0 (0)	2 (2.0)
Breast	5 (7.8)	2 (5.4)	7 (6.9)
Cervix	0 (0)	1 (2.7)	1 (1.0)
Chondrosarcoma	2 (3.1)	0 (0 0)	2 (2.0)
Fallopian tube	4 (6.3)	1 (2.7)	5 (5.0)
Glioblastoma	3 (4.7)	0 (0)	3 (3.0)
Gastric	1 (1.6)	0 (0)	1 (1.0)
Leiomyosarcoma	1 (1.6)	0 (0)	1 (1.0)
Mesothelioma	0 (0)	1 (2.7)	1 (1.0)
Non-small-cell lung cancer	1 (1.6)	0 (0)	1 (1.0)
Ovarian	33 (51.6)	23 (62.2)	56 (55.4)
Pancreatic	2 (3.1)	0 (0)	2 (2.0)
Peritoneal	1 (1.6)	1 (2.7)	2 (2.0)
Prostate	5 (7.8)	7 (18.9)	12 (11.9)
Small-cell lung cancer	3 (4.7)	1 (2.7)	4 (4.0)
Uterine	1 (1.6)	0 (0)	1 (1.0)

*ECOG* Eastern Cooperative Oncology Group.

^a^One patient with an ECOG status of 2 was incorrectly enrolled; however, this did not constitute a major protocol violation.

^b^The patient with an unknown primary tumour type had squamous cell carcinoma histology.

### Safety/tolerability profile

During BID dose-escalation (2.5–120 mg), all 45 patients experienced ≥1 AE (Supplementary Table S4). The most frequently reported AEs were nausea (64.4%), vomiting (35.6%), fatigue (33.3%), anaemia (33.3%) and diarrhoea (28.9%), with no clear dose effect (Table 2). Treatment-related AEs led to dose interruptions or dose reductions for 14 (31.1%) and three (6.7%) patients, respectively (Supplementary Table S4). Dose-limiting toxicities were observed in four of these 45 patients (*n* = 1, 40 mg; *n* = 1, 80 mg; *n* = 2, 120 mg) (Supplementary Table S4). The DLTs were Grade 2 nausea (across the 40- to 120-mg BID dosages) and Grade 2 anorexia and Grade 3 fatigue and paraesthesia (at the 120-mg BID dosage).

**Table 2:** Summary of adverse events of any grade occurring in >2 patients in the total BID dose-escalation cohort (*N* = 45).

BID dose-escalation cohort									
	2.5 mg (*n* = 4)	5.0 mg (*n* = 3)	10 mg (*n* = 3)	20 mg (*n* = 3)	40 mg (*n* = 6)	60 mg (*n* = 11)	80 mg (*n* = 10)	120 mg (*n* = 5)	Total (*N* = 45)
Nausea	3 (75.0)	0 (0.0)	1 (33.3)	2 (66.7)	5 (83.3)	6 (54.5)	8 (80.0)	4 (80.0)	29 (64.4)
Vomiting	1 (25.0)	0 (0.0)	1 (33.3)	2 (66.7)	5 (83.3)	4 (36.4)	2 (20.0)	1 (20.0)	16 (35.6)
Fatigue	1 (25.0)	1 (33.3)	0 (0.0)	3 (100.0)	2 (33.3)	2 (18.2)	4 (40.0)	2 (40.0)	15 (33.3)
Anaemia	1 (25.0)	0 (0.0)	1 (33.3)	0 (0.0)	2 (33.3)	4 (36.4)	4 (40.0)	3 (60.0)	15 (33.3)
Diarrhoea	0 (0.0)	1 (33.3)	1 (33.3)	2 (66.7)	2 (33.3)	4 (36.4)	1 (10.0)	2 (40.0)	13 (28.9)
Abdominal pain	1 (25.0)	0 (0.0)	1 (33.3)	2 (66.7)	0 (0.0)	3 (27.3)	2 (20.0)	0 (0.0)	9 (20.0)
Constipation	1 (25.0)	0 (0.0)	1 (33.3)	0 (0.0)	1 (16.7)	1 (9.1)	4 (40.0)	0 (0.0)	8 (17.8)
Upper respiratory tract infection	1 (25.0)	0 (0.0)	0 (0.0)	0 (0.0)	1 (16.7)	1 (9.1)	5 (50.0)	0 (0.0)	8 (17.8)
Urinary tract infection	0 (0.0)	0 (0.0)	0 (0.0)	1 (33.3)	0 (0.0)	3 (27.3)	2 (20.0)	2 (40.0)	8 (17.8)
Neutropenia	0 (0.0)	0 (0.0)	0 (0.0)	0 (0.0)	2 (33.3)	0 (0.0)	3 (30.0)	1 (20.0)	6 (13.3)
Ascites	1 (25.0)	0 (0.0)	2 (66.7)	0 (0.0)	1 (16.7)	0 (0.0)	1 (10.0)	0 (0.0)	5 (11.1)
Decreased appetite	0 (0.0)	0 (0.0)	0 (0.0)	0 (0.0)	0 (0.0)	1 (9.1)	2 (20.0)	2 (40.0)	5 (11.1)
Hypomagnesemia	0 (0.0)	0 (0.0)	1 (33.3)	1 (33.3)	0 (0.0)	0 (0.0)	2 (20.0)	0 (0.0)	4 (8.9)
Pyrexia	1 (25.0)	0 (0.0)	1 (33.3)	0 (0.0)	0 (0.0)	0 (0.0)	1 (10.0)	1 (20.0)	4 (8.9)
Headache	0 (0.0)	1 (33.3)	0 (0.0)	1 (33.3)	2 (33.3)	0 (0.0)	0 (0.0)	0 (0.0)	4 (8.9)
Back pain	0 (0.0)	0 (0.0)	0 (0.0)	0 (0.0)	2 (33.3)	1 (9.1)	0 (0.0)	1 (20.0)	4 (8.9)
Muscle spasms	1 (25.0)	1 (33.3)	0 (0.0)	0 (0.0)	0 (0.0)	1 (9.1)	1 (10.0)	0 (0.0)	4 (8.9)
Cough	0 (0.0)	0 (0.0)	1 (33.3)	0 (0.0)	0 (0.0)	1 (9.1)	1 (10.0)	1 (20.0)	4 (8.9)
Pleural effusion	0 (0.0)	1 (33.3)	0 (0.0)	0 (0.0)	0 (0.0)	2 (18.2)	0 (0.0)	1 (20.0)	4 (8.9)
Dyspnoea	0 (0.0)	0 (0.0)	1 (33.3)	1 (33.3)	0 (0.0)	1 (9.1)	0 (0.0)	0 (0.0)	3 (6.7)
Pain in extremity	0 (0.0)	0 (0.0)	0 (0.0)	1 (33.3)	0 (0.0)	1 (9.1)	0 (0.0)	1 (20.0)	3 (6.7)
Non-cardiac chest pain	0 (0.0)	0 (0.0)	0 (0.0)	0 (0.0)	0 (0.0)	1 (9.1)	1 (10.0)	1 (20.0)	3 (6.7)
Peripheral sensory neuropathy	0 (0.0)	1 (33.3)	0 (0.0)	1 (33.3)	0 (0.0)	1 (9.1)	0 (0.0)	0 (0.0)	3 (6.7)
Hypoalbuminemia	1 (25.0)	0 (0.0)	0 (0.0)	0 (0.0)	0 (0.0)	2 (18.2)	0 (0.0)	0 (0.0)	3 (6.7)
Hypophosphatemia	0 (0.0)	0 (0.0)	0 (0.0)	0 (0.0)	1 (16.7)	1 (9.1)	0 (0.0)	1 (20.0)	3 (6.7)
Increased AST	0 (0.0)	0 (0.0)	0 (0.0)	0 (0.0)	1 (16.7)	1 (9.1)	0 (0.0)	1 (20.0)	3 (6.7)

*AST* aspartate aminotransferase, *BID* twice daily.

Data presented as *n* (%).

Of the 101 total patients enrolled across the dose-escalation and dose-expansion phases, key non-haematologic AEs (all Grades and Grades ≥3, respectively) were nausea (69.3% and 4.0%), fatigue (48.5% and 3.0%), diarrhoea (32.7% and 2.0%), vomiting (31.7% and 1.0%), and increased alanine aminotransferase (11.9% and 5.0%) (Supplementary Table S5). Key haematologic AEs (all Grades and Grades ≥3) were anaemia (35.6% and 24.8%) and neutropenia (9.9% and 5.9%). Haematologic AEs led to dose reduction/interruption in 23.8% of patients (anaemia, 21.8%; neutropenia, 5.9%; and thrombocytopenia, 4.0%). Adverse events led to treatment discontinuation in six patients (5.9%) and were primarily gastrointestinal disorders (four patients experienced six AEs); there was one occurrence each of extradural haematoma, increased alanine aminotransferase, and paraesthesia that led to discontinuation (two patients experienced three AEs). A summary of commonly reported serious AEs is presented in the Supplemental Appendix.

### Pharmacokinetics and pharmacodynamics

**Fig. 2:**
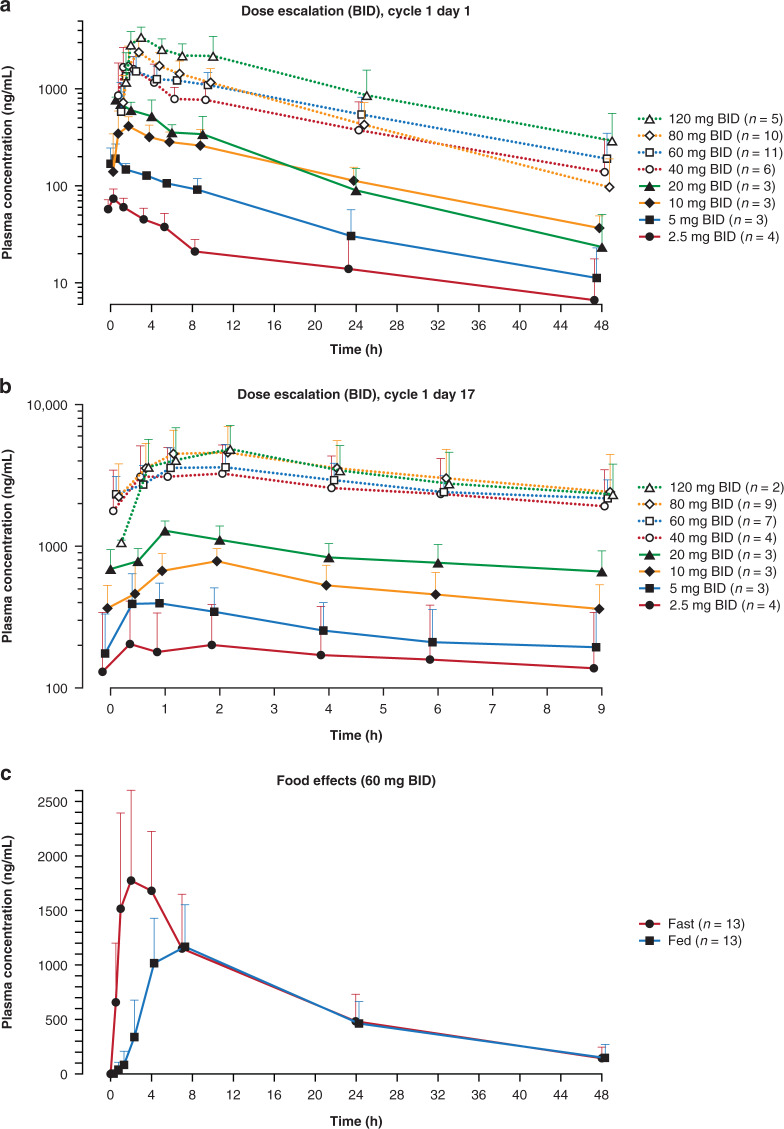
Mean concentration-time profiles with BID dosing. **a** With single dose on Cycle 1 day 1. **b** At steady state on Cycle 1 day 17. **c** Food effects with single dose. BID twice a day.

Across pamiparib BID dose-escalation levels (2.5–120 mg), a dose-dependent increase in exposure (Fig. 2a, b) and a dose-proportional increase in *C*_max_ and AUC_0-inf_ (Supplementary Table S6) was observed. In addition, pamiparib was rapidly absorbed, with a median *T*_max_ of 1 to 2 h (Supplementary Table S7); the geometric mean half-life of pamiparib at 60 mg BID was 13.5 h. Rates of pamiparib accumulation for AUC_0-9_ and *C*_max_ at 60 mg BID were 2.4 and 2.0, respectively, which are consistent with pamiparib’s half-life (Supplementary Table S8).

When administered with a high-fat breakfast, the rate of absorption of pamiparib was delayed, with median *T*_max_ prolonged to 7 h from 2 h (Fig. 2c) and AUC_0-inf_ and *C*_max_ reduced by 12% and 41%, respectively.

There was a dose-dependent increase in PAR inhibition in PBMCs from 2.5 mg to 10 mg BID; the inhibition was sustained at approximately 80% at pamiparib doses of 10 mg BID or higher (Supplemental Fig. 1A, B).

### Antitumor activity

Pamiparib demonstrated antitumor activity with BID dosing. In the BID efficacy-evaluable population (*n* = 77) across dose-escalation and dose-expansion phases, the confirmed ORR was 27.3% (95% CI, 17.7–38.6%) per RECIST v1.1 as assessed by the investigator (Table 3); confirmed CRs and PRs were observed in four (5.2%) and 17 (22.1%) patients, respectively. Thirty-seven patients (48.1%) had stable disease (SD), and the disease control rate was 75.3% (95% CI, 64.2–84.4). The median duration of response in the BID dosage group was 14.9 months (95% CI, 8.7–26.3). The best percent change from baseline in target lesion sum of product diameters by the best overall response in the overall BID and QD dose groups among patients in the efficacy-evaluable population with posttreatment assessments (*N* = 82) is shown in Supplemental Fig. 2A and B, respectively.

**Table 3:** Best overall response in the total BID dosage group.

Total BID dosage group		
	Overall efficacy-evaluable population (*n* = 77)	EOC efficacy-evaluable population (*n* = 51)
Best overall response, *n* (%)		
Complete response	4 (5.2)	4 (7.8)
Partial response	17 (22.1)	17 (33.3)
Stable disease	37 (48.1)	24 (47.1)
Progressive disease	11 (14.3)	2 (3.9)
Not evaluable	1 (1.3)	1 (2.0)
Not assessed^a^	7 (9.1)	3 (5.9)
Objective response rate, % (95% CI)^b^	27.3 (17.7–38.6)	41.2 (27.6–55.8)
Clinical benefit rate, % (95% CI)^c^	39.0 (28.1–50.8)	54.9 (40.3–68.9)
Disease control rate, % (95% CI)^d^	75.3 (64.2–84.4)	88.2 (76.1–95.6)

*BID* twice a day, *CI* confidence interval, *EOC* epithelial ovarian cancer.

^a^Patients in the efficacy-evaluable analysis set who discontinued before postbaseline tumour assessment due to disease progression or death are listed with a best overall response of not assessed.

^b^Objective response rate = complete response + partial response.

^c^Clinical benefit rate = complete response, partial response, or stable disease lasting at least 24 weeks without disease progression.

^d^Disease control rate = complete response, partial response, or stable disease as confirmed best response.

**Fig. 3:**
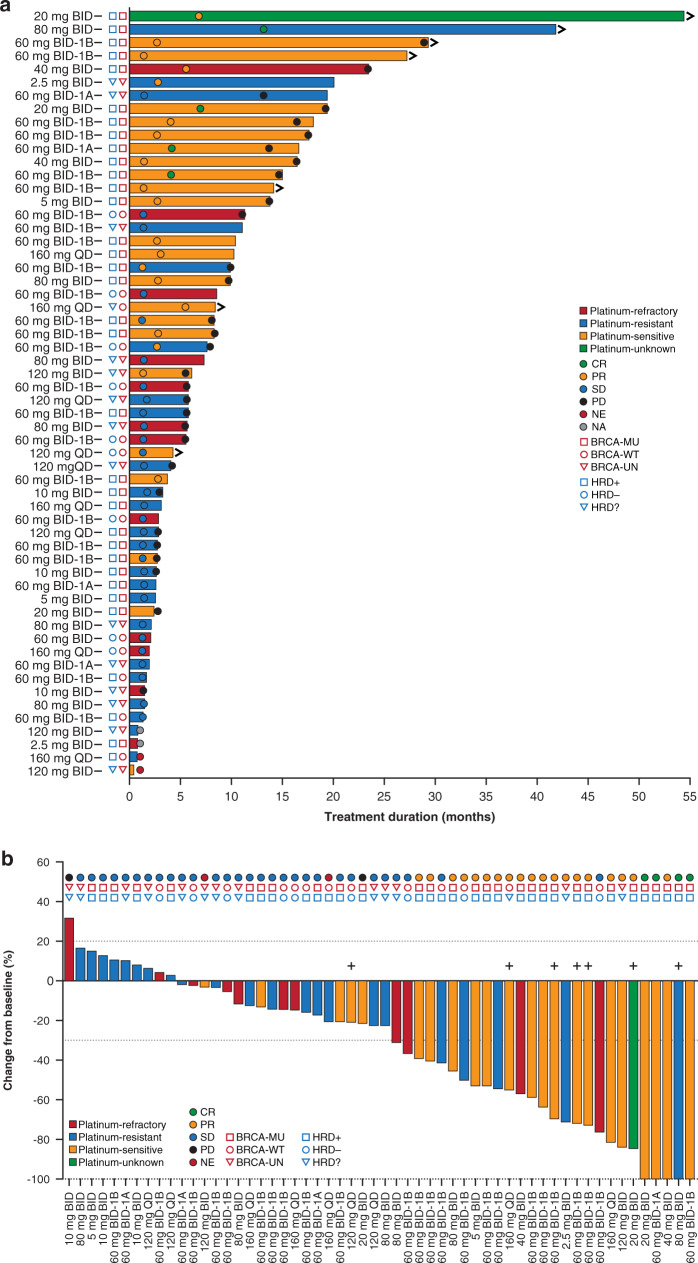
Duration of treatment and best percentage change from baseline in target lesions in patients with EOC. **a** Duration of treatment. **b** Best percentage change from baseline in target lesions. Symbols > in (**a**) and + in (**b**) denote the seven patients who continued pamiparib after closure of the study. BID twice a day, BRCA breast cancer susceptibility gene, CR complete response, EOC epithelial ovarian cancer, HRD homologous recombination deficiency, MU mutated, NA not applicable, NE not estimable, PD progressive disease, PR partial response, QD once a day, SD stable disease, UN unknown, WT wild-type.

All observed responses occurred in the total BID and QD EOC efficacy-evaluable population (*n* = 60). In the BID dosage group (*n* = 51), four patients achieved a confirmed CR (20 mg [*n* = 1], 60 mg [*n* = 2], and 80 mg [*n* = 1]) and 17 patients achieved a PR (Table 3). The duration of treatment for individual patients in the BID and QD EOC population is shown in Fig. 3a. Overall, an ORR of 41.2% (95% CI, 27.6–55.8) was observed and nearly half of the patients (*n* = 24) achieved SD; the disease control rate was 88.2% (95% CI, 76.1–95.6) and the clinical benefit rate was 54.9% (95% CI, 40.3–68.9). Objective responses with tumour reductions were observed in both platinum-sensitive and platinum-resistant cohorts, regardless of *BRCA* mutation and HRD status (Fig. 3b).

Responses observed in other populations are shown in Supplementary Tables S9 and  S10. Tumour response data for the CA-125-evaluable population (*n* = 12) and the PSA-evaluable population (*n* = 6) are presented in the Supplemental Appendix.

Results of subgroup analyses from the total BID and QD EOC efficacy-evaluable population demonstrated baseline disease characteristics, such as *BRCA* status, HRD status, and platinum sensitivity, may be associated with higher ORR (Supplementary Table S11). The increased response rates were observed among patients with a germline *BRCA* mutation (ORR, 66.7% [*n*/*N* = 18/60]), germline or somatic *BRCA* mutation (ORR, 61.3% [*n*/*N* = 31/60]), HRD-positive tumours (ORR, 55.9% [*n*/*N* = 34/60]), and platinum-sensitive disease (ORR, 75.0% (*n*/*N* = 24/60)). However, due to the small sample size, these data should be interpreted with caution.

### Progression-free survival

As of September 3, 2019, 60/95 (63.2%) efficacy-evaluable patients had either died (*n* = 10, 10.5%) or progressed (*n* = 50, 52.6%) (Supplementary Table S12). Median PFS for patients in the total BID and QD EOC efficacy-evaluable population was 8.3 months (95% CI, 5.45–13.67) and the 1- and 2-year event-free rates were estimated as 41.0% and 14.8%, respectively (Supplementary Table S13).

## Discussion

This FIH study of oral pamiparib monotherapy used a dose-escalation and dose-finding design to establish an RP2D of 60 mg BID and the MTD of 80 mg BID. Results showed that pamiparib was generally tolerated in this dose-escalation/dose-expansion study that enrolled patients with advanced solid tumours. Within the prespecified DLT assessment window, only non-haematological toxicities—primarily nausea—leading to dose interruption and dose reduction were observed in Cycle 1. In the total safety population, the percentage of patients who experienced haematological AEs in this study is similar to the percentages reported in other studies that also evaluate PARP inhibitors as monotherapy in advanced cancer. More specifically, anaemia is the most common Grade ≥3 AE associated with olaparib (17%), rucaparib (24.9% for anaemia/decreased haemoglobin), and niraparib (24%). These rates are in line with those observed for pamiparib in the current study (24.8%) and in the Phase 1 portion of the study (NCT03333915) conducted in patients in China (27%) [13–16]. In the separate Phase 3 trials of olaparib, rucaparib, and niraparib, which led to their approval in ovarian cancer, anaemia was also a commonly reported haematological AE (any Grade and Grade ≥3, respectively: niraparib, 50% and 25%; olaparib, 44% and 19%; rucaparib, 37% and 19%) [17–19]. Haematological AEs occur early after initiation of treatment with PARP inhibitors with recovery within a few months [20]. Nonclinical study results have shown that PARP2 plays an essential role in erythropoiesis, suggesting that anaemia is related to PARP2 inhibition [21]. PARP inhibition could also affect the folate pathway based on case reports of folate deficiency occurring within weeks of PARP inhibitor initiation in women with ovarian cancer [22].

The BID dosing of pamiparib 2.5 mg to 120 mg showed a dose-dependent increase in exposure with linear PK; the mean terminal half-life was approximately 13 h. Administration of pamiparib with food reduced the AUC and *C*_max_ by 12% and 41%, respectively. However, the reduction of AUC was not considered clinically relevant because it is within the variability of plasma exposure. The fact that PAR inhibition of approximately 80% was achieved and maintained in PBMCs at 10 mg BID or above also suggests that this magnitude of reduction in AUC and *C*_max_ after a high-fat breakfast is unlikely to change the extent of target inhibition in patients. These results indicate that patients may take pamiparib without regard to food.

Clinical benefit of pamiparib was associated with patient disease biomarker profile and sensitivity to platinum-based chemotherapy. High response rates observed in the current study among EOC patients with *BRCA*^*mut*^ (ORR, 66.7%), either germline or somatic *BRCA*^*mut*^ (ORR, 61.3%), HRD-positive (ORR, 55.9%), and platinum-sensitive disease (ORR, 75.0%) are notable and are indicative of EOC patient populations that may derive the most clinical benefit from pamiparib. Alongside the high response rates in *BRCA*^*mut*^ EOC patients, it is important to note nearly one-quarter of EOC patients with *BRCA* wild-type or unknown mutation status responded to pamiparib. These data are consistent with the efficacy observed in the Phase 1 study conducted in China (NCT03333915) in which patients with *BRCA*^mut^ and *BRCA* wild-type high-grade ovarian cancer who were refractory or resistant to platinum chemotherapy demonstrated an ORR of 25.0% (95% CI, 3.2–65.1) and a clinical benefit rate of 62.5% (95% CI, 24.5–91.5) in response to pamiparib treatment [13]. This is consistent with the suggestion that some cancers (notably ovarian) may still exhibit sensitivity to PARP inhibition even in the absence of *BRCA*^*mut*^ expression, but harbour other underlying defects in the homologous recombination repair pathway. Our findings are in line with the results of a Phase 2 study that showed an association of rucaparib’s clinical benefit with these clinical and molecular biomarkers [16]. In addition, patients in the Phase 2 rucaparib study who had *BRCA1/*2 wild-type status had mutations in non-*BRCA* homologous recombination genes, including *ATM*, *RAD51C* and *RAD51D*, which led to approval of rucaparib for the maintenance treatment of patients with ovarian cancer regardless of *BRCA1/2* status [23, 24].

A key objective of this study was to determine appropriate dosing for future clinical studies. Although both BID and QD dosing schedules were assessed in the current study, QD dosing was added as an amendment to the protocol with the purpose of exploring a convenient regimen for the future. Thus, the focus of the current study is outcomes from the BID dosing schedule as the study was not originally designed to further explore the QD regimen in expansion cohorts. In the BID dosage group of this study’s efficacy-evaluable population, the ORR was 27.3% (95% CI, 17.7–38.6%). The median duration of response in the BID dosage group was 14.9 months (95% CI, 8.7–26.3). All four CRs and 17 PRs occurred in the BID dosage group of the EOC efficacy-evaluable population and resulted in an ORR of 41.2% (95% CI, 27.6–55.8%). Two PRs occurred in the QD dosage group of the EOC population resulting in an ORR of 22.2% (95% CI, 2.8–60.0%). These initial results in our EOC efficacy-evaluable population are in line with Phase 2 results of other PARP inhibitors, which have been approved for use in patients with platinum-sensitive ovarian cancer [3, 15, 16]. In the overall populations of the Phase 2 studies, patients with *BRCA*^*mut*^ ovarian cancer who received prior platinum-based chemotherapy achieved an ORR of 31.1% (95% CI, 24.6–38.1) with olaparib, 28.0% (95% CI, 15.6–42.6) with niraparib, and 53.8% (95% CI, 43.8–63.5) with rucaparib [3, 15, 16].

Although anaemia was the most frequent AE reported in this study, nausea was the predominant DLT observed in four patients, two of which occurred at the 120-mg BID dose level; as such, the MTD was established as 80 mg BID. Haematological toxicities were not observed during the prespecified 21-day DLT assessment period; therefore, they were not factored into the RP2D determination. The RP2D was proposed as 60 mg BID because of its overall AE profile and lower incidence of nausea compared with 80 mg BID (54.5% at 60 mg versus 80.0% at 80 mg) as well as the clinical response observed throughout dose levels investigated. Based on the RP2D determined in this study, pamiparib 60 mg BID is currently being evaluated as a single agent in platinum-sensitive recurrent ovarian cancer (NCT03519230) and platinum-sensitive gastric cancer for first-line maintenance treatment (NCT03427814), and in combination with temozolomide in advanced solid tumours (NCT03150810) or with radiation and/or temozolomide in glioblastoma multiforme (NCT03150862). The safety profile and preliminary antitumour activity of pamiparib observed in this study serve as the basis for continued evaluation in patients with solid tumours. Ultimately, the safety profile, antitumour activity, and unique characteristics of pamiparib may increase its utility in the treatment of patients with various solid tumours.

### Reporting summary

Further information on research design is available in the Nature Research Reporting Summary linked to this article.

## Supplementary information

BJC Reporting Summary Checklist

Supplement-Clean Version

## Data Availability

On request, and subject to certain criteria, conditions and exceptions, BeiGene, Ltd., will provide access to individual de-identified participant data from BeiGene-sponsored global interventional clinical studies conducted for medicines (1) for indications that have been approved or (2) in programs that have been terminated. BeiGene will also consider requests for the protocol, data dictionary and statistical analysis plan. Data requests may be submitted to DataDisclosure@beigene.com.
