# Correction: Phase 1A/1B dose-escalation and -expansion study to evaluate the safety, pharmacokinetics, food effects and antitumor activity of pamiparib in advanced solid tumours

**DOI:** 10.1038/s41416-021-01671-9

**Published:** 2021-12-20

**Authors:** Jason D. Lickliter, Mark Voskoboynik, Linda Mileshkin, Hui K. Gan, Ganessan Kichenadasse, Kathy Zhang, Maggie Zhang, Zhiyu Tang, Michael Millward

**Affiliations:** 1 Nucleus Network, Melbourne, VIC Australia; 2 Central Clinical School, Monash University, Melbourne, VIC Australia; 3 Peter MacCallum Cancer Centre-East Melbourne, East Melbourne, VIC Australia; 4 Olivia Newton-John Cancer Wellness and Research Centre, Austin Hospital, Heidelberg, Melbourne, VIC Australia; 5 La Trobe University School of Cancer Medicine, Heidelberg, VIC Australia; 6 Department of Medicine, University of Melbourne, Heidelberg, VIC Australia; 7 Flinders Centre for Innovation in Cancer, Flinders Medical Centre, Bedford Park, SA Australia; 8 BeiGene USA, Inc., San Mateo, CA USA; 9 Linear Clinical Research & University of Western Australia, Nedlands, WA Australia

**Keywords:** Ovarian cancer, Drug development

Correction to: *British Journal of Cancer* 10.1038/s41416-021-01632-2, published online 18 November 2021

The article “Phase 1A/1B dose-escalation and -expansion study to evaluate the safety, pharmacokinetics, food effects and antitumor activity of pamiparib in advanced solid tumours”, written by Jason D. Lickliter, Mark Voskoboynik, Linda Mileshkin, Hui K. Gan, Ganessan Kichenadasse, Kathy Zhang, Maggie Zhang, Zhiyu Tang, Michael Millward, was originally published electronically on the publisher’s internet portal on 18 November 2021 without open access. With the author(s)’ decision to opt for Open Choice the copyright of the article changed on 2 December 2021 to © The Authors 2021and the article is forthwith distributed under a Creative Commons Attribution 4.0 International License, which permits use, sharing, adaptation, distribution and reproduction in any medium or format, as long as you give appropriate credit to the original author(s) and the source, provide a link to the Creative Commons licence, and indicate if changes were made. The images or other third party material in this article are included in the article’s Creative Commons licence, unless indicated otherwise in a credit line to the material. If material is not included in the article’s Creative Commons licence and your intended use is not permitted by statutory regulation or exceeds the permitted use, you will need to obtain permission directly from the copyright holder. To view a copy of this licence, visit http://creativecommons.org/licenses/by/4.0/

