# Supplementary material for: Phase 1A/1B dose-escalation and -expansion study to evaluate the safety, pharmacokinetics, food effects and antitumor activity of pamiparib in advanced solid tumours
Source: Br J Cancer. 2021 Nov 18;126(4):576–85. doi: 10.1038/s41416-021-01632-2 (PMC8854719; doi:10.1038/s41416-021-01632-2)
Supplement: Supplementary file 2 — Supplement-Clean Version [file 41416_2021_1632_MOESM2_ESM.docx]

**Phase 1A/1B Dose-Escalation and -Expansion Study to Evaluate the Safety, Pharmacokinetics, Food Effects, and Antitumor Activity of Pamiparib in Advanced Solid Tumors**

Jason D. Lickliter^1^, Mark Voskoboynik^2^, Linda Mileshkin^3^, Hui K. Gan^4^, Ganessan Kichenadasse^5^, Kathy Zhang^6^, Jang Yun^6^, Maggie Zhang^6^, Zhiyu Tang^6^, Song Mu^6^, Michael Millward^7^

**Supplemental Appendix**

**Methods**

*Patient Population*

All patients were required to have an Eastern Cooperative Oncology Group (ECOG) performance status of 0 or 1, and adequate organ function. Patients were not permitted to have previously received chemotherapy, biologic therapy, immunotherapy, or an investigational agent within five times the half-life of the agent or within 4 weeks of starting the study drug, whichever was longer. Patients who previously received therapeutic radiotherapy to target lesions were not eligible for inclusion in the study. Lesions that required palliative radiotherapy during the study were considered non-target lesions. Local palliative radiotherapy of non-target lesions within 21 days of study initiation was permitted as long as the patient had recovered from any associated adverse effects.

*Treatment Administration*

An example of a recommended United States Food and Drug Administration (US FDA) standard high-fat meal comprises two eggs fried in butter, two strips of bacon, two slices of toast with butter, 4 ounces of hash brown potatoes, and 8 ounces of whole milk. US FDA guidelines allow substitutions if the meal provides similar amounts of calories from protein (150), carbohydrate (250), and fat (500-600) with comparable meal volume.

*Assessments*

The PK profile of pamiparib was assessed based on blood samples collected at predose and 0.5, 1, 2, 4, 6, 9, 24, and 48 hours postdose on Days 1-3 and then at predose and 0.5, 1, 2, 4, 6, and 9 hours postdose on Day 17 of Cycle 1 in Phase 1A. Sparse PK samples were collected in phase 1B Part A. The food effect on the PK of pamiparib was assessed based on blood samples collected at predose and 0.5, 1, 2, 4, 7, 24, and 48 hours postdose on Day 1 and Day 6 of Cycle 1 in phase 1B Part B.

Time-matched blood samples (PK and PBMCs) were obtained on Day 1 (predose and 4 hours postdose) and Day 17 (4 hours postdose) of Cycle 1. The PAR levels in PBMCs were measured by a validated ELISA assay using a commercial PARP pharmacodynamic assay kit (Trevigen, Gaithersburg, MD). The pharmacodynamic activity at 4 hours postdose on Day 1 and predose on Day 17 were presented as percentage PAR inhibition from the baseline on Day 1 (predose).

*Statistical Methods*

Continuous variables were summarized by number, mean, standard deviation, median, Q1 (the median of the lower half of the data), Q3 (the median of the upper half of the data), and minimum/maximum values. Categorical variables were summarized by their frequency and percentage.

**Results**

*Safety/Tolerability Profile*

During dose escalation, all patients in the twice-daily (BID) dosage cohort (N=45) and the once-daily (QD) dosage cohort (N=19) experienced ≥1 adverse event (AE); 28 patients (62.2%) in the BID dosage cohort and 14 patients (73.7%) in the QD dosage cohort experienced a grade ≥3 AE (**Table S3**). The highest percentage of treatment-related grade ≥3 AEs occurred in the 120-mg BID dosage cohort (60.0%) and the 160-mg (63.6%) QD dosage cohort (**Table S3**).

In the QD dosage group (N=19), nausea (overall, 73.7%; 120 mg, 50.0%; 160 mg, 90.9%) was the most commonly reported AE followed by fatigue (overall, 68.4%; 120 mg, 75.0%; 160 mg, 63.6%) and diarrhea (overall, 47.4%; 120 mg, 50.0%; 160 mg, 45.5%). In the 19 patients enrolled in the QD dose-escalation phase, one patient who received pamiparib 160 mg experienced multiple events of nausea and vomiting during Cycle 1; the nausea persisted despite anti-emetic intervention, and of these events, grade 2 nausea was determined as the dose-limiting toxicity (DLT).

The maximum tolerated dose (MTD) of pamiparib QD dosing was determined as 160 mg based on one of 11 (9%) patients who experienced a DLT at that dosage; the recommended phase 2 dose (RP2D) was determined as 120 mg QD. Treatment-related AEs led to dose interruptions or dose reductions for 11 (57.9%) and one (5.3%) patient(s), respectively, in the QD dosage group (**Table S3**).

Of the 101 patients enrolled in the dose-escalation and dose-expansion phases, serious AEs were reported for 44 patients (43.6%), with the most common being small intestinal obstruction (5.0%), anemia (4.0%), nausea (4.0%), intestinal obstruction (4.0%), malignant gastrointestinal obstruction (3.0%), pleural effusion (3.0%), and pneumonia (3.0%). The 12 patients who experienced intestinal obstruction were in the EOC cohort; nine patients were platinum-resistant, two patients were platinum-sensitive, and one patient was platinum-refractory.

*Antitumor Activity*

In the BID dose-escalation group (N=44), the objective response rate (ORR) was 22.7% (95% CI, 11.5-37.8) per Response Evaluation Criteria in Solid Tumors (RECIST) v1.1 as assessed by the investigator (**Table S8**). There was no clear dose response with ORRs; response by dose should be interpreted with caution due to small sample sizes during dose escalation.

In the overall efficacy-evaluable population (n=95), the confirmed ORR was 24.2% (95% CI, 16.01%-34.08%) per RECIST v1.1 as assessed by the investigator; confirmed complete and partial responses (CRs and PRs) were observed in four (4.2%) and 19 (20%) patients, respectively. The confirmed ORR in the QD dosage group (n=18) was 11.1% (95% CI, 1.38-34.71) with no confirmed CRs and two (11.1%) confirmed PRs. In patients with epithelial ovarian cancer (EOC) who were evaluable for efficacy (N=60; BID, n=51, QD, n=9), four patients achieved a CR (6.7%) and 19 (31.7%) patients achieved a PR (**Table S9**), per RECIST v1.1, for an ORR of 38.3% (95% CI, 26.07-51.79). Half of the patients with EOC (n=30) achieved stable disease; the disease control rate was 88.3% (95% CI, 77.43-95.18) and the clinical benefit rate was 51.7% (95% CI, 38.39-64.77). In the QD dosage group (n=9), the ORR was 22.2% (95% CI, 2.81-60.01), the disease control rate was 88.9% (95% CI, 51.75-99.72), and the clinical benefit rate was 33.3% (95% CI, 7.49-70.07).

The GCIG CA-125 response rate was 83.3% (95% CI, 51.6-97.9) in the CA-125–evaluable population (n=12); median time to response was 1.38 months (range, 1.2-2.8).

No patients with mCRPC in phase 1B Part A were considered responders, as assessed by PCWG2 criteria. The PSA progression rate was 33.3% (95% CI, 4.3-77.7) in the PSA-evaluable population (n=6); median time to PSA progression was 4.2 months (range, 2.8-5.5).

**Figure S1. PAR Inhibition in PBMCs by Pamiparib Dosage on (A) Cycle 1 Day 1 and (B) Cycle 1 Day 17**


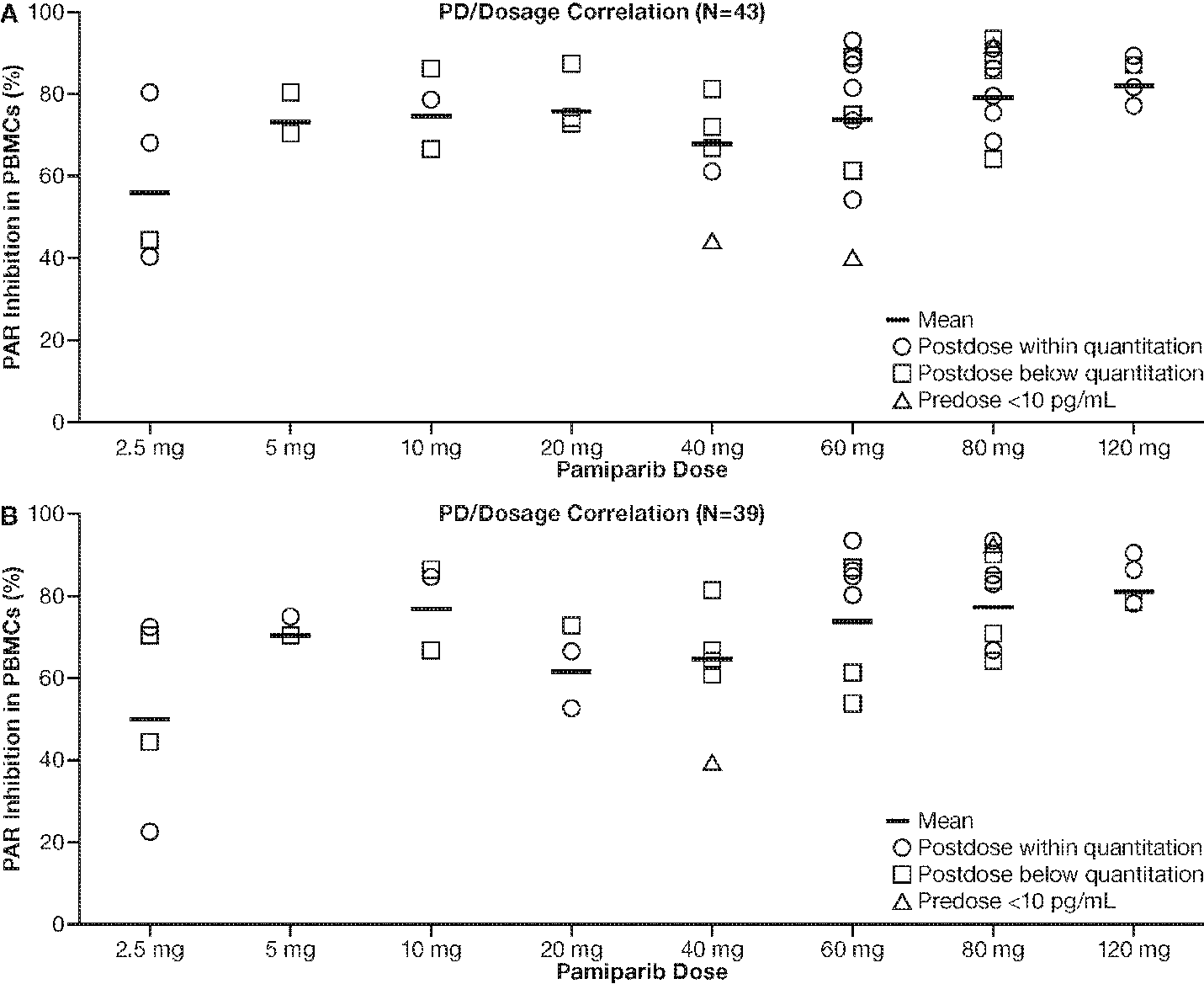


Abbreviations: PAR, poly(ADP-ribose); PBMC, peripheral blood mononuclear cell; PD, pharmacodynamics.

The pharmacodynamic activity at 4 hours postdose on Day 1 and Day 17 is presented as the percentage of PAR inhibition from the baseline on Day 1 (predose). □ Measured PAR of post-treatment was below of quantitation and the lower limit of quantitation was used to calculate PAR inhibition. The actual PAR inhibition is potentially greater than the calculated PAR inhibition. Δ The detected PAR level at baseline (predose sample) is below 10 pg/mL (close to the lower limit of quantitation). The actual PAR Inhibition is not accurate and excluded from the statistics.

**Figure S2. Best Percent Change From Baseline in Target Lesion Sum of Product Diameters by Best Overall Response During (A) Dose-Escalation and (B) Dose-Expansion Phases (Efficacy-Evaluable Population)**


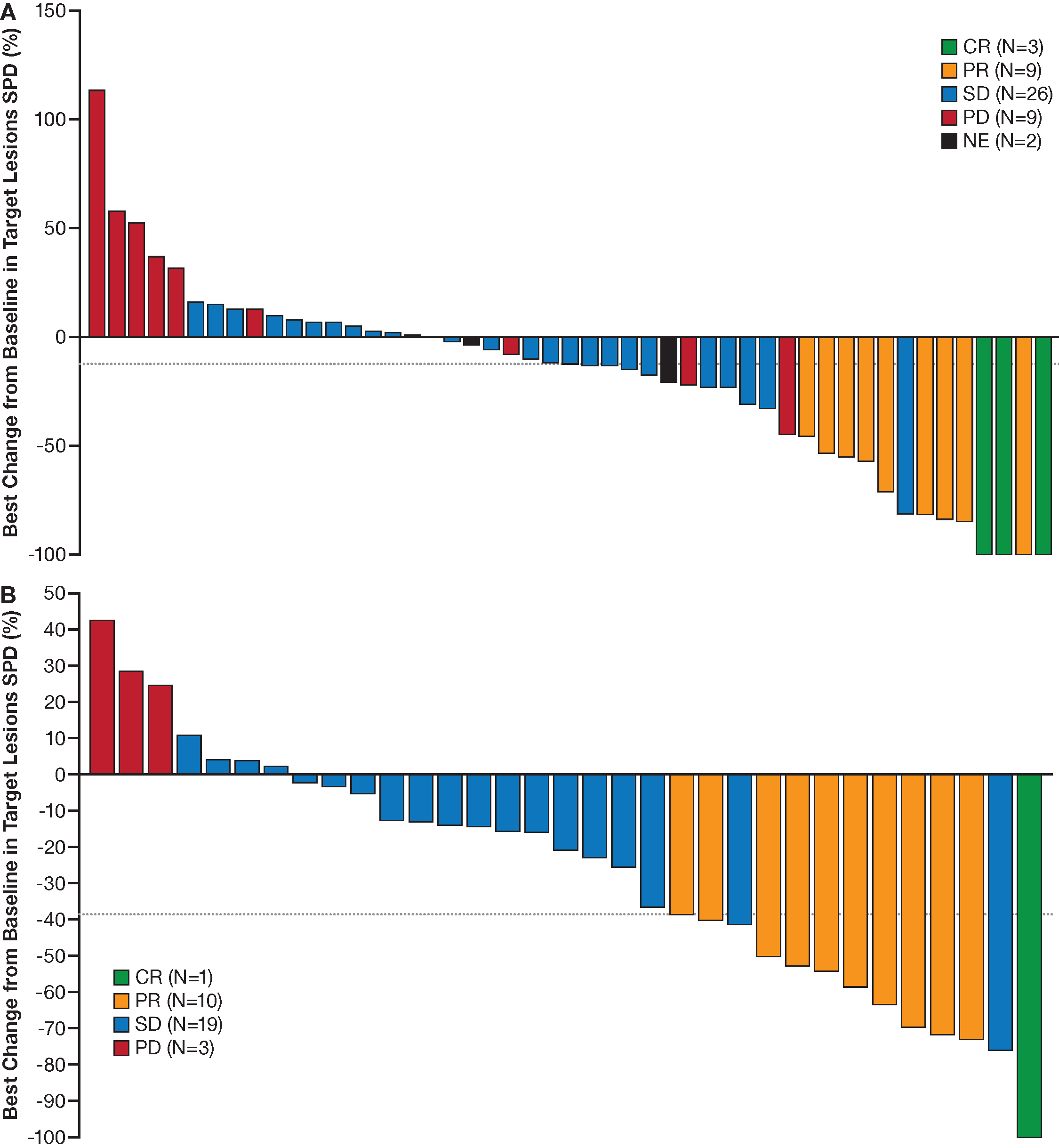


Abbreviations: CR, complete response; NE, not evaluable; PD, progressive disease; PR, partial response; SD, stable disease; SPD, sum of product diameters.

**Table S1. Criteria for Dose-Limiting Toxicities**

| **Non-hematologic** | - **Grade ≥3 nausea, vomiting, and diarrhea despite optimal supportive care** - **Any other clinically relevant grade ≥3 non-hematologic toxicity (excluding asymptomatic biochemical abnormalities that are not clinically significant and resolve to grade ≤2 in <7 days)** - **Persistent grade 2 toxicity, such as nausea, vomiting, and fatigue despite optimal standard medical therapy that in the opinion of the investigator prevents continuous dosing** - **Any toxicity grade which, in the judgment of the investigator or sponsor, is dose limiting** |
| --- | --- |

**Table S2. Analysis Populations**

|  | **Dose Escalation, n (%)** | | | **Dose Expansion, n (%)** | | | **Overall Population, n (%)** |
| --- | --- | --- | --- | --- | --- | --- | --- |
|  | **BID**  **(n=45)** | **QD**  **(n=19)** | **Total dose escalation**  **(n=64)** | **60 mg BID cohort**  **(n=24)** | **Food-effects**  **cohort**  **(n=13)** | **Total dose expansion**  **(n=37)** | **Total**  **(N=101)** |
| Safety population^a^ | 45 (100.0) | 19 (100.0) | 64 (100.0) | 24 (100.0) | 13 (100.0) | 37 (100.0) | 101 (100.0) |
| PK population^b^ | 45 (100.0) | 19 (100.0) | 64 (100.0) | 17 (70.8) | 13 (100.0) | 30 (81.1) | 94 (93.1) |
| PD population^c^ | 44 (97.8) | 0 (0.0) | 44 (68.8) | 0 (0.0) | 0 (0.0) | 0 (0.0) | 44 (43.6) |
| Efficacy-evaluable population^d^ | 44 (97.8) | 18 (94.7) | 62 (96.9) | 21 (87.5) | 12 (92.3) | 33 (89.2) | 95 (94.1) |
| Efficacy-evaluable population with EOC | 27 (60.0) | 9 (47.4) | 36 (56.3) | 16 (66.7) | 8 (61.5) | 24 (64.9) | 60 (59.4) |
| CA-125-evaluable population^e^ | 17 (37.8) | 8 (42.1) | 25 (39.1) | 12 (50.0) | 9 (69.2) | 21 (56.8) | 46 (45.5) |
| PSA-evaluable population^f^ | 0 (0.0) | 3 (15.8) | 3 (4.7) | 6 (25.0) | 0 (0.0) | 6 (16.2) | 9 (8.9) |

^a^ Safety population included all patients who received at least one dose of pamiparib.

^b^ PK population includes patients in Safety Analysis Set for whom valid pamiparib PK parameters can be estimated as of the data cutoff date.

^c^ PD population includes patients in Safety Analysis Set who had evaluable PD results available as of the data cutoff date.

^d^ Efficacy-evaluable population includes patients in the safety population who 1) had at least one evaluable postbaseline tumor assessment or 2) discontinued due to clinical disease progression or early death before tumor assessment. Patients in phase 1B without measurable disease at baseline per RECIST 1.1 were excluded.

^e^ CA-125-evaluable population includes patients with EOC who had a sample within 2 weeks prior to the first dose date that was at least twice the upper limit of the reference range.

^f^ PSA-evaluable population includes patients with metastatic castration-resistant prostate cancer who had a baseline PSA sample prior to the first dose date and at least one postbaseline PSA sample before date of new anticancer treatment.

Abbreviations: BID, twice a day; CA-125, carcinoma antigen-125; EOC, epithelial ovarian cancer; PD, pharmacodynamics; PK, pharmacokinetics; PSA, prostate-specific antigen; QD, once a day; RECIST, Response Evaluation Criteria in Solid Tumors.

**Table S3. Patient Demographics in the EOC Population**

|  | **Dose-Escalation (n=38)** | | **Dose-Expansion (n=25)** | **Total**  **(N=63)** |
| --- | --- | --- | --- | --- |
| **Median time from initial diagnosis to study entry, year (range)** | 3.41 (0.5-11.6) | | 2.57 (0.6-20.4) | 2.84 (0.5-20.4) |
| **Type of solid tumor, n (%)** |  | |  |  |
| Ovarian | 33 (86.8) | | 23 (92.0) | 56 (88.9) |
| Fallopian | 4 (10.5) | | 1 (4.0) | 5 (7.9) |
| Peritoneal | 1 (2.6) | | 1 (4.0) | 2 (3.2) |
| **Germline *BRCA* status, n (%)** |  | |  |  |
| Mutant | 5 (13.2) | | 13 (52.0) | 18 (28.6) |
| Wild-type | 9 (23.7) | | 12 (48.0) | 21 (33.3) |
| Unknown | 24 (63.2) | | 0 (0) | 24 (38.1) |
| ***BRCA* status (germline or somatic), n (%)** |  |  | |  |
| Mutant | 17 (44.7) | 14 (56.0) | | 31 (49.2) |
| Wild-type | 5 (13.2) | 10 (40.0) | | 15 (23.8) |
| Unknown | 16 (42.1) | 1 (4.0) | | 17 (27.0) |
| **HRD status, n (%)** |  |  | |  |
| Positive | 18 (47.4) | 16 (64.0) | | 34 (54.0) |
| Negative | 3 (7.9) | 8 (32.0) | | 11 (17.5) |
| Unknown | 17 (44.7) | 1 (4.0) | | 18 (28.6) |
| **Germline *BRCA* mutant or HRD-positive, n (%)** |  |  | |  |
| Yes | 18 (47.4) | 16 (64.0) | | 34 (54.0) |
| No | 20 (52.6) | 9 (36.0) | | 29 (46.0) |
| **Median number of prior regimens (range)** | 3 (1-15) | 3 (1-7) | | 3 (1-15) |
| **Platinum sensitivity status, n (%)** |  |  | |  |
| Refractory | 6 (15.8) | 6 (24.0) | | 12 (19.0) |
| Resistant | 18 (47.4) | 8 (32.0) | | 26 (41.3) |
| Sensitive | 14 (36.8) | 11 (44.0) | | 25 (39.7) |
| Abbreviations: BRCA, breast cancer susceptibility gene; HRD, homologous recombination deficiency. | | | | |

**Table S4. Summary of Treatment-Emergent Adverse Events During Dose Escalation**

|  | **BID Dose-Escalation Cohort** | | | | | | | | | **QD Dose-Escalation Cohort** | | |
| --- | --- | --- | --- | --- | --- | --- | --- | --- | --- | --- | --- | --- |
| **n (%)** | **2.5 mg**  **(n=4)** | **5.0 mg**  **(n=3)** | **10 mg**  **(n=3)** | **20 mg**  **(n=3)** | **40 mg**  **(n=6)** | **60 mg**  **(n=11)** | **80 mg**  **(n=10)** | **120 mg (n=5)** | **Total**  **(N=45)** | **120 mg (n=8)** | **160 mg (n=11)** | **Total (N=19)** |
| **Treatment-emergent AE** | 4 (100.0) | 3 (100.0) | 3 (100.0) | 3 (100.0) | 6 (100.0) | 11 (100.0) | 10 (100.0) | 5 (100.0) | 45 (100.0) | 8 (100.0) | 11 (100.0) | 19 (100.0) |
| Grade 3 or higher | 3 (75.0) | 1 (33.3) | 2 (66.7) | 1 (33.3) | 3 (50.0) | 9 (81.8) | 4 (40.0) | 5 (100.0) | 28 (62.2) | 5 (62.5) | 9 (81.8) | 14 (73.7) |
| Serious | 3 (75.0) | 2 (66.7) | 2 (66.7) | 1 (33.3) | 2 (33.3) | 9 (81.8) | 3 (30.0) | 3 (60.0) | 25 (55.6) | 2 (25.0) | 4 (36.4) | 6 (31.6) |
| Leading to death | 0 (0.0) | 0 (0.0) | 0 (0.0) | 0 (0.0) | 0 (0.0) | 3 (27.3) | 1 (10.0) | 0 (0.0) | 4 (8.9) | 0 (0.0) | 0 (0.0) | 0 (0.0) |
| Leading to treatment discontinuation | 0 (0.0) | 0 (0.0) | 0 (0.0) | 0 (0.0) | 1 (16.7) | 0 (0.0) | 0 (0.0) | 2 (40.0) | 3 (6.7) | 0 (0.0) | 2 (18.2) | 2 (10.5) |
| Leading to dose modification | 1 (25.0) | 1 (33.3) | 1 (33.3) | 3 (100.0) | 4 (66.7) | 8 (72.7) | 5 (50.0) | 4 (80.0) | 27 (60.0) | 6 (75.0) | 7 (63.6) | 13 (68.4) |
| Leading to dose interruption | 1 (25.0) | 1 (33.3) | 1 (33.3) | 3 (100.0) | 4 (66.7) | 8 (72.7) | 5 (50.0) | 4 (80.0) | 27 (60.0) | 6 (75.0) | 7 (63.6) | 13 (68.4) |
| Leading to dose reduction | 0 (0.0) | 0 (0.0) | 0 (0.0) | 1 (33.3) | 0 (0.0) | 0 (0.0) | 1 (10.0) | 1 (20.0) | 3 (6.7) | 1 (12.5) | 0 (0.0) | 1 (5.3) |
| **Treatment-related AE** | 3 (75.0) | 2 (66.7) | 0 (0.0) | 3 (100.0) | 6 (100.0) | 7 (63.6) | 9 (90.0) | 5 (100.0) | 35 (77.8) | 6 (75.0) | 11 (100.0) | 17 (89.5) |
| Grade 3 or higher | 0 (0.0) | 0 (0.0) | 0 (0.0) | 1 (33.3) | 2 (33.3) | 1 (9.1) | 3 (30.0) | 3 (60.0) | 10 (22.2) | 3 (37.5) | 7 (63.6) | 10 (52.6) |
| Serious | 0 (0.0) | 0 (0.0) | 0 (0.0) | 0 (0.0) | 0 (0.0) | 1 (9.1) | 1 (10.0) | 1 (20.0) | 3 (6.7) | 0 (0.0) | 1 (9.1) | 1 (5.3) |
| Leading to death | 0 (0.0) | 0 (0.0) | 0 (0.0) | 0 (0.0) | 0 (0.0) | 0 (0.0) | 0 (0.0) | 0 (0.0) | 0 (0.0) | 0 (0.0) | 0 (0.0) | 0 (0.0) |
| Leading to treatment discontinuation | 0 (0.0) | 0 (0.0) | 0 (0.0) | 0 (0.0) | 0 (0.0) | 0 (0.0) | 0 (0.0) | 2 (40.0) | 2 (4.4) | 0 (0.0) | 2 (18.2) | 2 (10.5) |
| Leading to dose modification | 0 (0.0) | 0 (0.0) | 0 (0.0) | 1 (33.3) | 3 (50.0) | 2 (18.2) | 5 (50.0) | 3 (60.0) | 14 (31.1) | 4 (50.0) | 7 (63.6) | 11 (57.9) |
| Leading to dose interruption | 0 (0.0) | 0 (0.0) | 0 (0.0) | 1 (33.3) | 3 (50.0) | 2 (18.2) | 5 (50.0) | 3 (60.0) | 14 (31.1) | 4 (50.0) | 7 (63.6) | 11 (57.9) |
| Leading to dose reduction | 0 (0.0) | 0 (0.0) | 0 (0.0) | 1 (33.3) | 0 (0.0) | 0 (0.0) | 1 (10.0) | 1 (20.0) | 3 (6.7) | 1 (12.5) | 0 (0.0) | 1 (5.3) |
| Dose-limiting toxicity | 0 (0.0) | 0 (0.0) | 0 (0.0) | 0 (0.0) | 1 (16.7)^a^ | 0 (0.0) | 1 (10.0)^b^ | 2 (40.0)^c^ | 4 (8.9) | 0 (0.0) | 1 (9.1)^d^ | 1 (5.3) |

^a^Grade 2 nausea.

^b^Grade 2 nausea.

^c^1. Grade 2 nausea and grade 2 anorexia; 2. Grade 2 nausea, grade 3 fatigue, and grade 3 paresthesia.

^d^Grade 2 nausea.

Abbreviations: AE, adverse event; BID, twice daily; QD, once daily.

**Table S5. Adverse Events of Any Grade (≥10%) and of Grade ≥3**

| **Summary of AEs** | **Dose Escalation and Dose Expansion**  **(N=101)** | |
| --- | --- | --- |
| **AEs of any grade (≥10%) and grade ≥3** | **All Grades** | **Grade ≥3** |
| Nausea | 70 (69.3) | 4 (4.0) |
| Fatigue | 49 (48.5) | 3 (3.0) |
| Anemia | 36 (35.6) | 25 (24.8) |
| Diarrhea | 33 (32.7) | 2 (2.0) |
| Vomiting | 32 (31.7) | 1 (1.0) |
| Decreased appetite | 23 (22.8) | 0 |
| Constipation | 21 (20.8) | 0 |
| Abdominal pain | 17 (16.8) | 1 (1.0) |
| Urinary tract infection | 14 (13.9) | 1 (1.0) |
| Upper respiratory tract infection | 12 (11.9) | 0 |
| Headache | 12 (11.9) | 0 |
| Increased alanine aminotransferase | 12 (11.9) | 5 (5.0) |
| Neutropenia | 10 (9.9) | 6 (5.9) |
| Increased aspartate aminotransferase | 10 (9.9) | 3 (3.0) |

Data presented as n (%).

Abbreviation: AE, adverse event.

**Table S6. Dose Proportionality for Pamiparib BID Dosing in the Dose-Escalation Phase**

| **Parameter** | **n** | **Intercept (95% CI)** | **Slope (95% CI)** |
| --- | --- | --- | --- |
| **Single dose** | | | |
| AUC_0-inf_ (h*ng/mL) | 44 | 5.97 (5.36-6.57) | 1.04 (0.88-1.20) |
| C_max_ (ng/mL) | 45 | 3.66 (3.34-3.99) | 0.96 (0.87-1.04) |
| **Steady state** | | | |
| AUC_last, ss_ (h*ng/mL) | 35 | 6.10 (5.52-6.68) | 0.95 (0.79-1.11) |
| C_max, ss_ (ng/mL) | 35 | 4.47 (3.98-4.95) | 0.90 (0.77-1.04) |

Abbreviations: AUC_0-inf_, area under the plasma concentration-time curve from 0 to infinity; AUC_last_, area under the plasma concentration-time curve up to the last measurable concentration; BID, twice daily; CI, confidence interval; C_max_, maximum observed plasma concentration; ss, steady state.

**Table S7.** **Summary of** **BID and QD Dose Pharmacokinetic Parameters of Pamiparib**

|  |  |  | **Single Dose (Cycle 1 Day 1)** | | | | | | | **Steady State (Cycle 1 Day 17)** | | |
| --- | --- | --- | --- | --- | --- | --- | --- | --- | --- | --- | --- | --- |
| **Regimen** | **Dose (mg)** | **N** | **C_max_ (ng/ml)** | **T_max_ (hr)** | **AUC_0-9_ (ng/ml*hr)** | **AUC_0-inf_ (ng/ml*hr)** | **t_1/2_**  **(hr)** | **Vz/F**  **(mL)** | **CL/F**  **(mL/hr)** | **C_max_ (ng/ml)** | **T_max_ (hr)** | **AUC_0-9_ (ng/ml*hr)** |
| **BID** | 2.5 | 4 | 74.5 (26.4) | 1.01  (0.53, 1.03) | 384.7  (33.7) | 586.1  (34.8) | 12.03  (7.08, 33.87) | 52404.1  (18.9) | 4265.3  (34.8) | 181.7  (91.2) | 0.54  (0.50, 1.93) | 931.3  (147.3) |
|  | 5 | 3 | 193.1 (38.0) | 1.00  (0.58, 2.00) | 1099.2  (15.3) | 2430.8  (49.5) | 11.01  (8.30, 17.25) | 32661.1  (8.1) | 2056.9  (49.5) | 404.2  (46.6) | 0.53  (0.50, 1.00) | 2117.9  (61.8) |
|  | 10 | 3 | 412.0 (31.2) | 2.00  (1.07, 2.00) | 2576.0  (38.5) | 7357.3  (35.9) | 14.08  (13.30, 14.56) | 27611.7  (35.9) | 1359.2  (35.9) | 656.8  (40.8) | 1.05  (1.00, 2.15) | 4327.2  (39.6) |
|  | 20 | 3 | 782.7 (9.4) | 0.53  (0.48, 1.00) | 4134.9  (29.9) | 7841.8  (58.7) | 8.53  (5.79, 11.91) | 31370.2  (18.2) | 2550.4  (58.7) | 1267.6  (19.9) | 1.00  (0.98, 1.02) | 7512.3  (31.6) |
|  | 40 | 6 | 1754.7 (44.3) | 1.04  (0.88, 2.00) | 8954.8  (44.4) | 21899.0  (85.8) | 11.76  (6.24, 24.37) | 30996.1  (37.4) | 1826.6  (85.8) | 2940.9  (74.1) | 1.50  (0.50, 2.00) | 18297.5  (99.4) |
|  | 60 | 11 | 1881.4 (27.0) | 2.00  (0.98, 9.00) | 10403.7  (29.2) | 32004.5  (55.4) | 13.50  (7.14, 24.61) | 36508.5  (22.1) | 1874.7  (55.4) | 3832.4  (44.8) | 2.00  (0.92, 8.92) | 25218.6  (39.9) |
|  | 80 | 10 | 2317.8 (43.0) | 1.98  (1.00, 2.03) | 13213.0  (41.5) | 28708.5  (56.0) | 9.58  (6.21, 14.29) | 38527.5  (35.4) | 2786.6  (56.0) | 4352.3  (56.3) | 1.08  (0.92, 4.00) | 25276.6  (62.3) |
|  | 120 | 5 | 3586.5 (26.4) | 1.95  (1.00, 2.02) | 21101.2  (33.4) | 52012.3  (85.3) | 10.91  (5.40, 17.58) | 36310.8  (26.4) | 2307.1  (85.3) | 4532.3  (54.0) | 2.00  (2.00, 2.00) | 26862.7  (66.3) |
| **QD** | 120 | 8 | 3752.8 (41.1) | 2.00  (1.00, 6.00) | 21725.1  (33.3) | 68952.9  (43.3) | 14.29  (9.83, 30.30) | 31373.9  (13.9) | 1740.3  (43.3) | 6059.1  (27.7) | 2.00  (0.97, 4.00) | 36558.4  (32.4) |
|  | 160 | 11 | 5336.0 (18.7) | 2.00  (0.93, 3.93) | 31820.5  (24.3) | 101966.6  (44.8) | 15.25  (10.43, 24.13) | 34526.2  (31.0) | 1569.1  (44.8) | 6872.7  (32.8) | 2.00  (1.00, 4.02) | 45264.4  (26.4) |

Abbreviations: AUC_0-9_, area under the plasma concentration-time curve from 0 to 9 hours; AUC_0-inf_, area under the plasma concentration-time curve from 0 to infinity; BID, twice daily; CL/F, apparent clearance; C_max_, maximum observed plasma concentration; QD, once a day; t_1/2_, elimination half-life; T_max_, time to reach C_max_; V_z_/F, apparent volume of distribution during terminal phase.

**Table S8. Pamiparib Accumulation Rate**

| **Parameter** | **Dosing Frequency** | **Dose** | **n** | **Accumulation Ratio** | **(95% CI)** |
| --- | --- | --- | --- | --- | --- |
| AUC_0-9_ (h*ng/mL) | BID | 2.5 mg | 4 | 2.42 | (1.57, 3.74) |
|  |  | 5 mg | 3 | 1.93 | (1.17, 3.18) |
|  |  | 10 mg | 3 | 1.68 | (1.02, 2.78) |
|  |  | 20 mg | 3 | 1.82 | (1.10, 3.00) |
|  |  | 40 mg | 4 | 1.86 | (1.20, 2.87) |
|  |  | 60 mg | 5 | 2.37 | (1.61, 3.50) |
|  |  | 80 mg | 8 | 2.07 | (1.52, 2.81) |
|  |  | 120 mg | 2 | 1.56 | (0.84, 2.88) |
|  | QD | 120 mg | 7 | 1.61 | (1.16, 2.23) |
|  |  | 160 mg | 7 | 1.47 | (1.06, 2.04) |
| C_max_ (ng/mL) | BID | 2.5 mg | 4 | 2.44 | (1.63, 3.64) |
|  |  | 5 mg | 3 | 2.09 | (1.32, 3.32) |
|  |  | 10 mg | 3 | 1.59 | (1.00, 2.53) |
|  |  | 20 mg | 3 | 1.62 | (1.02, 2.57) |
|  |  | 40 mg | 4 | 1.60 | (1.07, 2.39) |
|  |  | 60 mg | 7 | 1.99 | (1.47, 2.69) |
|  |  | 80 mg | 9 | 1.96 | (1.50, 2.56) |
|  |  | 120 mg | 2 | 1.54 | (0.87, 2.72) |
|  | QD | 120 mg | 7 | 1.55 | (1.10, 2.18) |
|  |  | 160 mg | 8 | 1.27 | (0.92, 1.75) |

Abbreviations: AUC_0-9_, area under the plasma concentration-time curve from 0 to 9 hours; BID, twice daily; CI, confidence interval; C_max_, maximum observed plasma concentration; QD, once a day

**Table S9. Best Overall Response by BID Dosage Cohort Based on Investigators’ Assessment per RECIST v1.1 in the Efficacy-Evaluable Population (Dose-Escalation Phase)**

|  | **BID Dose-Escalation Cohort** | | | | | | | | |
| --- | --- | --- | --- | --- | --- | --- | --- | --- | --- |
|  | **2.5 mg**  **(n=4)** | **5.0 mg**  **(n=3)** | **10 mg**  **(n=3)** | **20 mg**  **(n=3)** | **40 mg**  **(n=6)** | **60 mg**  **(n=11)** | **80 mg**  **(n=9)** | **120 mg (n=5)** | **Total**  **(N=44)** |
| **Best overall response, n (%)** | | | | | | | | | |
| Complete response | 0 (0.0) | 0 (0.0) | 0 (0.0) | 1 (33.3) | 0 (0.0) | 1 (9.1) | 1 (11.1) | 0 (0.0) | 3 (6.8) |
| Partial response | 1 (25.0) | 1 (33.3) | 0 (0.0) | 1 (33.3) | 2 (33.3) | 0 (0.0) | 1 (11.1) | 1 (20.0) | 7 (15.9) |
| Stable disease | 0 (0.0) | 1 (33.3) | 2 (66.7) | 0 (0.0) | 4 (66.7) | 5 (45.5) | 5 (55.6) | 1 (20.0) | 18 (40.9) |
| Progressive disease | 1 (25.0) | 0 (0.0) | 1 (33.3) | 1 (33.3) | 0 (0.0) | 3 (27.3) | 1 (11.1) | 1 (20.0) | 8 (18.2) |
| Not evaluable | 0 (0.0) | 0 (0.0) | 0 (0.0) | 0 (0.0) | 0 (0.0) | 0 (0.0) | 0 (0.0) | 1 (20.0) | 1 (2.3) |
| Not assessed^a^ | 2 (50.0) | 1 (33.3) | 0 (0.0) | 0 (0.0) | 0 (0.0) | 2 (18.2) | 1 (11.1) | 1 (20.0) | 7 (15.9) |
| **Objective response rate, % (95% CI)^b^** | 25.0  (0.63-80.59) | 33.3  (0.84-90.57) | 0.0  (0.00-70.76) | 66.7  (9.43-99.16) | 33.3  (4.33-77.72) | 9.1  (0.23-41.28) | 22.2  (2.81-60.01) | 20.0  (0.51-71.64) | 22.7  (11.47-37.84) |
| **Clinical benefit rate, % (95% CI)^c^** | 25.0  (0.63-80.59) | 33.3  (0.84-90.57) | 0.0  (0.00-70.76) | 66.7  (9.43-99.16) | 33.3  (4.33-77.72) | 18.2  (2.28-51.78) | 44.4  (13.70-78.80) | 20.0  (0.51-71.64) | 29.5  (16.76-45.20) |
| **Disease control rate, % (95% CI)^d^** | 25.0  (0.63-80.59) | 66.7  (9.43-99.16 | 66.7  (9.43-99.16) | 66.7  (9.43-99.16) | 100.0  (54.07-100.00) | 54.5  (23.38-83.25) | 77.8  (39.99-97.19) | 40.0  (5.27-85.34) | 63.6  (47.77-77.59) |

^a^Patients in the efficacy-evaluable analysis set who discontinued before postbaseline tumor assessment due to disease progression or death are listed with a best overall response of not assessed.

^b^Objective response rate = complete response + partial response.

^c^Clinical benefit rate = complete response, partial response, or stable disease lasting at least 24 weeks without disease progression.

^d^Disease control rate = complete response, partial response, or stable disease as confirmed best response.

Abbreviations: BID, twice a day; CI, confidence interval; RECIST, Response Evaluation Criteria in Solid Tumors.

**Table S10. Best Overall Response by Dosage Group Based on Investigators’ Assessment per RECIST v1.1 in the Efficacy-Evaluable Population With Epithelial Ovarian Cancer (N=60)**

|  | **Total BID Dosage Group (n=51)** | | **Total QD Dosage Group (n=9)** | **Total**  **(N=60)** |
| --- | --- | --- | --- | --- |
| **Best overall response, n (%)** | | | | |
| Complete response | 4 (7.8) | | 0 (0) | 4 (6.7) |
| Partial response | 17 (33.3) | | 2 (22.2) | 19 (31.7) |
| Stable disease | 24 (47.1) | | 6 (66.7) | 30 (50.0) |
| Progressive disease | 2 (3.9) | | 0 (0) | 2 (3.3) |
| Not evaluable | 1 (2.0) | | 1 (11.1) | 2 (3.3) |
| Not assessed^a^ | 3 (5.9) | | 0 (0) | 3 (5.0) |
| **Objective response rate, % (95% CI)^b^** | **41.2**  **(27.58-55.83)** | | **22.2**  **(2.81-60.01)** | **38.3**  **(26.07-51.79)** |
| **Clinical benefit rate, % (95% CI)^c^** | **54.9**  **(40.34-68.87)** | **33.3**  **(7.49-70.07)** | | **51.7**  **(38.39-64.77)** |
| **Disease control rate, % (95% CI)^d^** | **88.2**  **(76.13-95.56)** | **88.9**  **(51.75-99.72)** | | **88.3**  **(77.43-95.18)** |
| ^a^Patients in the efficacy-evaluable analysis set who discontinued before postbaseline tumor assessment due to disease progression or death are listed with a best overall response of ‘not assessed.’  ^b^Objective response rate = complete response + partial response.  ^c^Clinical benefit rate = complete response, partial response, or stable disease lasting at least 24 weeks without disease progression.  ^d^Disease control rate = complete response, partial response, or stable disease as confirmed best response.  Abbreviations: BID, twice a day; CI, confidence interval; QD, once a day; RECIST, Response Evaluation Criteria in Solid Tumors. | | | | |

**Table S11. Objective Response Rates by Patient Subgroup in the Efficacy-Evaluable Population With Epithelial Ovarian Cancer**

| **Subgroups, ORR, % (95% CI) [n/N]** | **Total BID Dosage Group (n=51)** | **Total QD Dosage Group**  **(n=9)** | **Total**  **(N=60)** |
| --- | --- | --- | --- |
| **Age** | | | |
| <65 | 42.1 (26.31-59.18)  [38/51] | 20.0 (0.51-71.64)  [5/9] | 39.5 (24.98-55.59)  [43/60] |
| ≥65 | 38.5 (13.86-68.42)  [13/51] | 25.0 (0.63-80.59)  [4/9] | 35.3 (14.21-61.67)  [17/60] |
| **Baseline ECOG performance status** | | | |
| 0 | 47.8 (26.82-69.41)  [23/51] | 0.0 (0.00-97.50)  [1/9] | 45.8 (25.55-67.18)  [24/60] |
| ≥1 | 35.7 (18.64-55.93)  [28/51] | 25.0 (3.19-65.09)  [8/9] | 33.3 (18.56-50.97)  [36/60] |
| **Solid tumor stage** | | | |
| 3 | 53.6 (33.87-72.49)  [28/51] | 0.0 (0.00-52.18)  [5/9] | 45.5 (28.11-63.65)  [33/60] |
| 4 | 23.8 (8.22-47.17)  [21/51] | 50.0 (1.26-98.74)  [2/9] | 26.1 (10.23-48.41)  [23/60] |
| **Germline *BRCA* status** | | | |
| Mutant | 73.3 (44.90-92.21)  [15/51] | 33.3 (0.84-90.57)  [3/9] | 66.7 (40.99-86.66)  [18/60] |
| Wild-type/Unknown | 27.8 (14.20-45.19)  [36/51] | 16.7 (0.42-64.12)  [6/9] | 26.2 (13.86-42.04)  [42/60] |
| **Germline or somatic *BRCA* status** | | | |
| Mutant | 64.3 (44.07-81.36)  [28/51] | 33.3 (0.84-90.57)  [3/9] | 61.3 (42.19-78.15)  [31/60] |
| Wild-type/Unknown^a^ | 13.0 (2.78-33.59)  [23/51] | 16.7 (0.42-64.12)  [6/9] | 13.8 (3.89-31.66)  [29/60] |
| **HRD status** | | | |
| Positive | 60.0 (40.60-77.34)  [30/51] | 25.0 (0.63-80.59)  [4/9] | 55.9 (37.89-72.81)  [34/60] |
| Negative/Unknown | 14.3 (3.05-36.34)  [21/51] | 20.0 (0.51-71.64)  [5/9] | 15.4 (4.36-34.87)  [26/60] |
| **Platinum-sensitivity status^b^** | | | |
| Sensitive | 76.2 (52.83-91.78)  [21/51] | 66.7 (9.43-99.16)  [3/9] | 75.0 (53.29-90.23)  [24/60] |
| Resistant | 21.1 (6.05-45.57)  [19/51] | 1. (0.00-52.18)   [5/9] | 16.7 (4.74-37.38)  [24/60] |
| Refractory | 9.1 (0.23-41.28)  [11/51] | 1. (0.00-97.50)   [1/9] | 8.3 (0.21-38.48)  [12/60] |
| ^a^13 patients had *BRCA* wild-type status; 14 patients had *BRCA* unknown status.  ^b^One patient had unknown platinum sensitivity status.  Abbreviations: BID, twice a day; BRCA, breast cancer susceptibility gene; CI, confidence interval; ECOG, Eastern Cooperative Oncology Group; HRD, homologous recombination deficiency; ORR, objective response rate; QD, once daily; RECIST, Response Evaluation Criteria in Solid Tumors.  Efficacy-evaluable population includes patients in the safety population who had measurable disease at baseline per RECIST 1.1, and had at least one evaluable post-baseline tumor assessment or discontinued due to clinical disease progression or early death prior to tumor assessment. n is the number of patients with a complete response or a partial response. | | | |

**Table S12. Progression-Free Survival Based on Investigators’ Assessment per RECIST v1.1 (Total Efficacy-Evaluable Population)**

| **Progression-Free Survival** | **Total BID Dosage Group (n=77)** | **Total QD Dosage Group (n=18)** | **Total**  **(N=95)** |
| --- | --- | --- | --- |
| Events, n (%) | 49 (63.6) | 11 (61.1) | 60 (63.2) |
| Death | 7 (9.1) | 3 (16.7) | 10 (10.5) |
| Disease progression | 42 (54.5) | 8 (44.4) | 50 (52.6) |
| Censored, n (%) | 28 (36.4) | 7 (38.9) | 35 (36.8) |
| ≥2 consecutive missed assessments | 2 (2.6) | 0 (0) | 2 (2.1) |
| New anticancer therapy received before event | 1 (1.3) | 0 (0) | 1 (1.1) |
| No disease progression or death | 20 (26.0) | 7 (38.9) | 27 (28.4) |
| No postbaseline assessment | 5 (6.5) | 0 (0) | 5 (5.3) |
| Median months (95% CI)^a^ | 6.9 (4.14-9.89) | 4.9 (3.22-12.32) | 5.5 (4.14-8.31) |
| Event-free rate, % (95% CI)^b^ |  |  |  |
| 1 year | 34.0 (21.72-46.58) | 30.1 (8.73-55.34) | 32.7 (21.73-44.18) |
| 2 years | 12.2 (4.52-24.01) | 0.0 (NE-NE) | 11.0 (4.13-21.80) |
| ^a^Median was estimated by the Kaplan-Meier method; 2-sided 95% CI was estimated using the Brookmeyer and Crowley method.  ^b^Event-free rates were estimated by the Kaplan-Meier method with 95% CIs estimated using the Greenwood formula.  Abbreviations: BID, twice a day; CI, confidence interval; NE, not estimable; QD, once a day; RECIST, Response Evaluation Criteria in Solid Tumors. | | | |

**Table S13. Progression-Free Survival Based on Investigators’ Assessment per RECIST v1.1 (EOC Efficacy-Evaluable Population)**

| **Progression-Free Survival** | **Total BID Dosage Group (n=51)** | **Total QD Dosage Group (n=9)** | **Total**  **(N=60)** |
| --- | --- | --- | --- |
| **Events, n (%)** | 32 (62.7) | 7 (77.8) | 39 (65.0) |
| Death | 6 (11.8) | 3 (33.3) | 9 (15.0) |
| Disease progression | 26 (51.0) | 4 (44.4) | 30 (50.0) |
| **Censored, n (%)** | 19 (37.3) | 2 (22.2) | 21 (35.0) |
| ≥2 consecutive missed assessments | 2 (3.9) | 0 (0) | 2 (3.3) |
| No disease progression or death | 15 (29.4) | 2 (22.2) | 17 (28.3) |
| No postbaseline assessment | 2 (3.9) | 0 (0) | 2 (3.3) |
| **Median months (95% CI)^a^** | 9.9 (5.49-14.62) | 4.9 (1.84-NE) | 8.3 (5.45-13.67) |
| **Event-free rate, % (95% CI)^b^** | | | |
| 1 year | 45.6 (29.51-60.25) | NE (NE-NE) | 41.0 (26.93-54.59) |
| 2 years | 16.4 (5.98-31.33) | NE (NE-NE) | 14.8 (5.46-28.41) |
| ^a^Median was estimated by the Kaplan-Meier method; 2-sided 95% CI was estimated using the Brookmeyer and Crowley method.  ^b^Event-free rates were estimated by the Kaplan-Meier method with 95% CIs estimated using the Greenwood formula.  Abbreviations: BID, twice a day; CI, confidence interval; EOC, epithelial ovarian cancer; NE, not estimable; QD, once a day; RECIST, Response Evaluation Criteria in Solid Tumors. | | | |
